# Intramuscular Injection and Complex Regional Pain Syndrome Development After “Harmless” Procedures

**DOI:** 10.7759/cureus.9393

**Published:** 2020-07-25

**Authors:** Ryan Babineau, Richard Alweis

**Affiliations:** 1 Medicine, University of Rochester, Rochester, USA; 2 Internal Medicine, University of Rochester School of Medicine and Dentistry/Unity Health System, Rochester, USA

**Keywords:** complex regional pain syndrome, crps, intramuscular injection

## Abstract

Complex regional pain syndrome (CRPS) is a puzzling pain condition, typically occurring after trauma. Incidents as innocent as IV injection and vaccine administration may serve as the impetus for an as yet poorly understood cascade of neuro-inflammation and somatosensory changes. A condition with variable degrees of psychological involvement, disability, and purely clinical diagnostic criteria, the diagnosis is often significantly delayed before effective treatment is begun, decreasing the likelihood of success. A 58-year-old female initially presented with painless peripheral edema of the left arm without erythema or loss of function, with later bilateral development of multiple domains of symptoms consistent with CRPS. Plausible initiation events in this instance were an IV insertion in the left cubital fossa and vaccination into the right deltoid. Delay in diagnosis is common but utilization of the Budapest Criteria for diagnosis and prompt treatment improves outcomes.

## Introduction

Peripheral nerve injury after injection is a common, though usually transient, phenomenon. Sciatic neuropathy is the most commonly reported, but most injections, including vaccinations, carry some degree of risk [1]. Development of complex regional pain syndrome (CRPS) after an injection, however, is uncommon, and its variable manifestations may delay the diagnosis and subsequent treatment of the syndrome for significant periods of time [1-3]. CRPS symptoms have been recognized by multiple names over the course of its study. The Budapest Criteria were established to attempt to standardize the nomenclature and diagnostic criteria of CRPS. Reflex sympathetic dystrophy (RSD) is now identified as CRPS type 1, and Causalgia is now recognized as CRPS type 2 [4]. With evolving standards of identification and management, CRPS is a challenge for practicing physicians. We present the case of a patient who developed CRPS after receiving a vaccination against shingles, in which the variable presentation and time course of the condition led to a delay in diagnosis.

## Case presentation

A 58-year-old female with a history of hypothyroidism, impaired fasting glucose, nonalcoholic fatty liver disease, and stress urinary incontinence presented with left antecubital fossa pain and swelling at an IV placement site. She had undergone an abdominal and pelvic CT scan with contrast 48 hours earlier during an abdominal pain evaluation. The scan itself was unremarkable, but the insertion site became progressively more swollen and painful. She denied any trauma to the area other than the IV insertion. Her medications at the time of presentation were levothyroxine and premarin cream. She denied alcohol and tobacco usage. Her social history and family history were otherwise unremarkable. She was diagnosed with a superficial thrombophlebitis, and supportive therapy with heat and nonsteroidal anti-inflammatories were recommended.

Approximately one month later, she received her first dose of adjuvanted recombinant zoster vaccine in the right deltoid, followed two months later with the second dose, administered to the same deltoid. One month after this second injection, she presented with three weeks of painless soft tissue swelling of the right arm without erythema or loss of function. Complaints of right arm swelling were not corroborated by findings on physical examination, but swelling was apparent on the left arm at the site of the previous phlebitis. Ten days later, the patient reported unchanged swelling, as well as new right forearm tingling without pain. At this time a diagnosis of injection neuritis was given. Within four weeks, the patient presented again, with reports of new pain and swelling in the right antecubital fossa and pain in the right arm, in addition to the previous tingling. Gabapentin 300 mg thrice daily (later increased to 600 mg thrice daily) was prescribed and ultrasound of the right antecubital region was unremarkable. 

Over the course of four weeks, her pain and tingling sensations worsened, despite taking gabapentin. The patient now described constant tingling and burning associated with hypersensitivity to touch. On physical examination, there were skin color changes and swelling, but no obvious changes in sweat patterns. A diagnosis of CRPS type 2 was made. Gabapentin was discontinued due to reports of blurred vision, and duloxetine 30 mg was started. The patient was referred to desensitization therapy. A left antecubital ultrasound was performed, but was unremarkable. After no significant benefit was observed after two months on gabapentin, a right stellate ganglion block was performed with bupivacaine. Per the patient, the block provided only 50% relief for 48 hours. Bilateral arm pain, swelling and tingling, as well as spread of these symptoms to the anterior chest wall continued.

## Discussion

The CRPS type 1 accounts for the majority of cases, occurring without direct nerve damage, while CRPS type 2 strictly follows a direct nerve injury. The Budapest Criteria for the diagnosis of CRPS have established four distinct categories (sensory; vasomotor; sudomotor/edema; motor dysfunction and/or trophic changes), and presence of all four categories of signs and symptoms achieves sensitivity and specificity of 0.86 and 0.75, respectively [5]. They have also demonstrated increased inter-rater reliability between physicians [3-6]. However, no test is considered by all to be the “Gold Standard” in the diagnosis of CRPS [5-7]. More than 50,000 cases of CRPS are diagnosed per year, and it is assumed that many more are missed because physicians are not familiar with its presentation [8].

Common epidemiological features of CRPS shared by the patient include female gender and having upper distal limb symptoms, including allodynia and hyperalgesia [6]. Trauma, including fractures and surgeries, is commonly associated with CRPS diagnosis; however, the identification is purely clinical, as no lab testing or radiographs have been deemed pathognomonic, and the only notable traumas to this patient were an IV insertion and the two vaccinations [4, 6]. The patient’s timeline of symptoms aligns with the characteristic disease process, where an initial, “warm” phase is observed over the first few weeks after the inciting event, characteristic of inflammatory signs. The latter, “cold”, chronic phase, usually manifests months afterward. Typical complaints include skin changes, e.g., coloration and sweat patterns, and, more persistent hyperalgesia, which may generalize to other parts of the body unaffected by any antecedent trauma [6]. 

The variable presentations of the syndrome are hypothesized to be due to multifactorial changes in sensitization, neuronal dysregulation, and autonomic abnormalities [4]. There is a clinically significant risk of CRPS manifesting in another extremity if it occurred in another limb, as did our patient, with an odds ratio of greater than 1000 for re-development of CRPS after a second insult anywhere in the body, which implies a central process [9]. Contralateral spread is twice as common as ipsilateral, with diagonal advancement being quite rare [10]. Diffusion of symptoms is often spontaneous, further implicating a central process. 

The pathophysiology of CRPS is not fully elucidated, but four major components are believed to be involved: trauma, pain processing, autonomic dysfunction, and immune dysfunction [11]. Symptoms extending the innervation zone of an individual nerve are brought on by the cascade initiated by the trauma, leading to the dynamic, multifaceted manifestation of the disease [11]. Expanding literature indicates a possible genetic component of disease for immune and specific receptor sensitivities. The immune aspect of CRPS remains the least understood, showing inconsistent relationships in clinical studies [6,11]. As central nervous involvement expands with disease progression, it is believed that plasticity adjustments in primary motor cortex could play a role in hypersensitivity, jerk responses, and dystonia [6]. It has been hypothesized that the intricate network of somatosensation areas and cognition can lead to hyperalgesia via constitutive nociceptive activation. This change with CRPS may lead to smaller somatosensation areas in cortical tissue and be triggered even by the thought of movement [6]. Afferent and efferent signaling in the dorsal ganglion activates a cascade involving TNF-α for neurogenic inflammation [6]. Sympathetic activation can manifest in color change, temperature change, as well as hyper- and hypohidrosis. Autonomic changes differ in warm and cold stages of the condition [4, 7]. First, initial norepinephrine release wanes, leading to increased blood flow. Later, in the cold stage, increased catecholamine sensitivity promotes vasoconstriction and decreased blood flow [6]. Increased expression of adrenoreceptors on nociceptive fibers impacts the coupling of sympathetic activation and pain sensitivity in CRPS patients [6]. Validation of biomarkers involved in pathogenesis has the potential to improve diagnostic and remediation approaches by minimizing the subjectivity of current criteria [12]. 

Injection trauma is not a novel initiator of CRPS. Penetrating trauma can impact nerves and create inflammatory processes. Vaccine side effects are generally localized injection neuritis from the direct trauma, mass effect from the injected material, or, more rarely, caused by a direct toxic effect of the injected material, though CRPS has been reported [13]. Penetration trauma is more than likely the impetus for CRPS onset, as there is insufficient data claiming that the viral antigens of the injectant play a role, which was heavily analyzed for human papillomavirus [2,14]. 

The interaction between CRPS and psychological factors is highly complex. Sufferers often experience a cyclical pattern of pain, emotional stress, and a vegetative state where disuse can augment symptoms [8]. This cycle leads to prolonged disease, but not necessarily onset, and the chronic pain may generate depression rather than the converse [3]. Anxiety-related catecholamine release may increase nociception and adrenergic symptoms. Somatosensation and perception influence pain, as higher anxiety, anger, and depression correlated with increased pain days and preoperative anxiety also correlated with greater pain levels in certain subjects, though noncausal [8]. Among those with CRPS, people with allodynia are more distressed than others, possibly triggering their entrance into cyclic distress and avoidance [8]. Current literature does not robustly support any psychological predictors of CRPS, but does endorse poor coping mechanisms and large psychological burden to affect pain perceptions [4, 6, 15-16]. Pain intensity and anxiety correlated with disability and distress in CRPS patients, further complicating care [15]. Post-traumatic stress disorder (PTSD) is found more frequently in those with CRPS than in the general population [16]. Maladaptive coping strategies are related to PTSD, offering another complex avenue for patient symptoms to spiral. Psychological treatment of CRPS is yet to be comprehensively explored in randomized control trials, but concurrent treatment for psychological and physiological symptoms is the best current practice [3,15]. 

Current treatment modalities are broad due to the complexity of the disease. Aggressive, timely management is imperative for delivering positive CRPS outcomes. Common examples include physical therapy, neuropathic pain medication, anti-inflammatories, bisphosphonates, sympathetic nerve blocks, and direct neural stimulation [4,17]. Sympathetic blocks are often insufficient for most patients, and epidural clonidine may reveal adverse effects in addition to benefits [7]. Immunoglobulin infusion clinical trials were not found to be effective in CRPS management, bringing the autoimmune aspect of the disease into question [18]. Preliminary evidence indicates advantages to earlier introduction of neuromodulation if other modalities fail [17]. A multi-disciplinary pain clinic is often needed to coordinate the pharmacological, physical therapy, and procedural needs to recover limb functionality; however, prognosis is poor when patients are chronically symptomatic [6]. 

Poorly understood causes and occurrence after minor traumas that may not be recalled by the patient are a part of what makes diagnosis and management of CRPS so difficult for healthcare providers. Many of the missed cases are attributed to psychogenic issues, especially when treatments are not successful initially [8]. In this case, the patient’s condition may have been spurred by something as commonplace as an IV, or a shingles vaccine, which was perceived as harmless. Iatrogenic neuritis and neuropathy are not uncommon after injection and they should be added to the differential after a patient presents with pain seemingly out of proportion to examination findings [19-20]. Some suggest in-service training for staff using techniques for reducing the risk of serious complications, emphasizing site identification and proper hand placement [1]. A recent study found that many nurses were not explicitly taught in training about nerve sites relevant to injection [1]. Increased education for physicians using the biopsychosocial model can help treat new neuroinflammatory symptoms, and possibly avoid wastebasket diagnosis [8]. Further investigation into the mechanism of disease development is required to successfully provide evidence-based treatments.

## Conclusions

Complex regional pain syndrome may vary greatly in terms of presenting symptoms, many of which are subjective. Seemingly benign procedures have the potential to initiate a cascade of events, which result in life-altering pain. The CRPS etiology and progression are often not considered in such cases. Moreover, providers may achieve improved outcomes from familiarizing themselves with the new Budapest Criteria in hopes of rapid diagnosis and treatment.
